# Exploring nanobioceramics in wound healing as effective and economical alternatives

**DOI:** 10.1016/j.heliyon.2024.e38497

**Published:** 2024-09-26

**Authors:** Hanan Adnan Shaker Al-Naymi, Mastafa H. Al-Musawi, Marjan Mirhaj, Hamideh Valizadeh, Arefeh Momeni, Amir Mohammad Danesh Pajooh, Mina Shahriari-Khalaji, Fariborz Sharifianjazi, Ketevan Tavamaishvili, Nafise Kazemi, Saeideh Salehi, Ahmadreza Arefpour, Mohamadreza Tavakoli

**Affiliations:** a Department of Chemistry, College of Education for Pure Science/Ibn Al-Haitham, University of Baghdad, Baghdad, Iraq; b Department of Clinical Laboratory Science, College of Pharmacy, Mustansiriyah University, Baghdad, Iraq; c Department of Materials Engineering, Isfahan University of Technology, Isfahan, 84156-83111, Iran; d Department of tissue engineering and regenerative medicine, Faculty of advanced technologies in medicine, Iran university of medical sciences, Tehran, Iran; e Department of Biomedical Engineering, Amirkabir University of Technology, Tehran, Iran; f Department of Life Science Engineering, Faculty of New Sciences and Technologies, University of Tehran, Tehran, Iran; g State Key Laboratory for Modification of Chemical Fibers and Polymer Materials, College of Materials Science and Engineering, Donghua University, Shanghai, 201620, China; h Center for Advanced Materials and Structures, School of Science and Technology, The University of Georgia, 0171, Tbilisi, Georgia; i Department of Civil Engineering, School of Science and Technology, The University of Georgia, 0171, Tbilisi, Georgia; j Georgian American University, School of Medicine, 10 Merab Aleksidze Str., Tbilisi, 0160, Georgia; k Advanced Materials Research Center, Department of Materials Engineering, Najafabad Branch, Islamic Azad University, Najafabad, Iran

**Keywords:** Angiogenesis, Antibacterial, Bioceramics, Inorganic ions, Wound treatment

## Abstract

Wound healing is a sophisticated process for which various treatment methods have been developed. Bioceramics with the ability to release inorganic ions in biological environments play a crucial role in cellular metabolism and exhibit bactericidal activity, contributing to numerous physiological processes. Their multifaceted roles in biological systems highlight their significance. The release of different metallic ions from bioceramics enables the repair of both hard and soft tissues. These ions may be effective in cell motility, proliferation, differentiation, adhesion, angiogenesis, and antibiosis. Unlike conventional medications, the bioactivity and antibacterial properties of bioceramics are typically not associated with side effects or bacterial resistance. Bioceramics are commonly recognized for their capcity to facilitate the healing of hard tissues due to their exceptional mechanical properties. In this review, we first explore wound treatment and its prevalent methods, and subsequently, we discuss the application of three primary categories of bioceramics-oxide ceramics, silicate-based ceramics, and calcium-phosphate ceramics-in the context of wound treatment. This review introduces bioceramics as a cost-effective and efficient alternative for wound repair. Our aim is to inspire researchers to incorporate bioceramics with other biomaterials to achieve enhanced, economical, expedited, and safer wound healing.

## Graphical abstract

**Figure:**
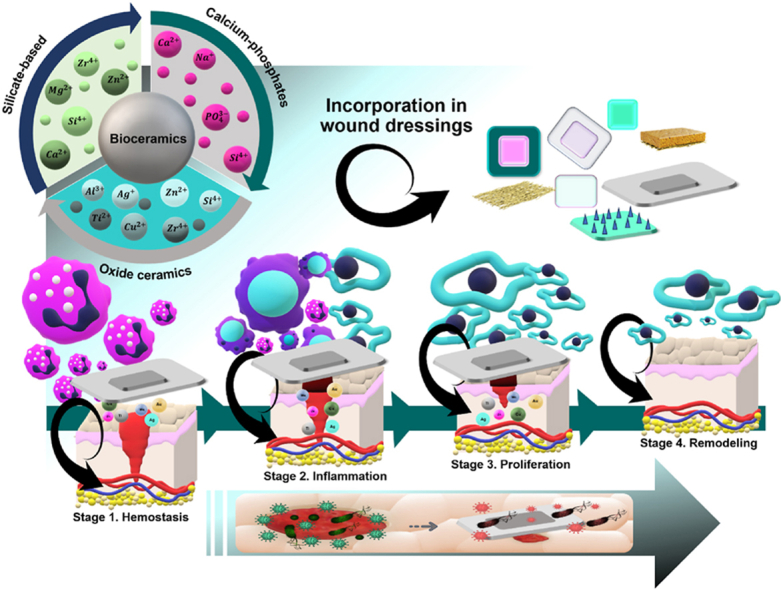

## 1 Introduction

As the body's largest and outermost tissue, the skin serves as its primary defense and responds to structural damage by initiating the wound healing process. While the skin possesses inherent self-healing capabilities, wounds that extend into the dermis and subcutaneous layers often exhibit limited self-repair capacity, necessitating therapeutic interventions for effective healing [[1], [2], [3], [4]]. In cases where wound healing is prolonged, leading to chronic conditions, the risk of infection increases, and in severe instances, it may even threaten the patient's life. Various treatment modalities are available for severe skin injuries, including skin transplantation, stem cell/cell therapy, platelet therapy, instrumental-based therapy, and the application of wound dressings. Although wound dressings are widely employed due to their efficiency and ease of application, they may not be sufficient for achieving optimal treatment outcomes. To address this challenge, the development of novel wound dressings capable of loading and releasing growth factors, drugs, and diverse ions to induce antibacterial activity or stimulate accelerated healing is currently underway [[5], [6], [7], [8]].

Wound dressings as carriers in direct contact with the wound site can play an important role in the targeted delivery of therapeutics. Many researchers have integrated growth factors and drugs into wound dressings to promote tissue regeneration, aiming to enhance overall wound healing outcomes. However, critical challenges associated with the use of growth factors include their short half-life, susceptibility to denaturation, and high cost [9]. Conversely, many drugs induce side effects within the human body. For instance, non-steroidal anti-inflammatory drugs, which are effective in reducing inflammation and pain, may hinder the natural wound healing process by exerting an anti-proliferative effect on blood vessels and skin cells. Moreover, steroid drugs such as dexamethasone possess anti-inflammatory and immunosuppressive properties, however, their use can delay wound healing [10]. Many common antibiotics such as mupirocin [11,12], gentamicin [13,14], vancomycin [15], ciprofloxacin [16] and tetracycline [17] may exhibit cytotoxic effects and thus interfere with wound healing. In addition, the bactericidal efficacy of many antibiotics may be significantly reduced due to bacterial resistance [18].

In light of the drawbacks and limitations associated with the use of growth factors and drugs, there is an urgent need for a viable, cost-effective alternative. Among bioactive materials, bioceramics with the ability to release essential ions have high therapeutic potential and at the same time create minimal side effects. Bioceramics are amorphous or crystalline ceramic materials designed for deployment within the human body, tissue repair or organ replacement. Historically, it was reported that these materials have been utilized for medical applications since calcium (Ca) sulfate was first applied in 975 AD for bone repair [19]. Bioceramics are primarily known for their applications in hard tissue repair and replacement, and they are less frequently studied for soft tissue regeneration applications. Nevertheless, excellent cytocompatibility, release of various ions, biochemical regulation of the microenvironment, and facilitation of intercellular signaling position bioceramics as materials with high potential for soft tissue regeneration [20]. The three primary classifications of bioceramics (calcium-phosphate ceramics, silicate-based ceramics, and oxide ceramics) are regarded as promising candidates for applications in wound healing, attributed to their distinctive features such as antibacterial activity, cell stimulation, and angiogenic potential.

Our objective is to conduct a comprehensive review of previous studies focused on the application of these bioceramics in the context of skin regeneration and wound healing. In contrast to many prior reviews on bioceramics, this overview aims to present these materials not only as effective but also as cost-efficient alternatives for wound repair.

## 2 Wound treatment

Wounds can developed due to wear, cuts, burns, diseases, accidents and surgeries, leading to the destruction of the natural anatomical relationship of skin tissue [21]. The wound healing process is intricate, dynamic and complicated, involving four main stages: homeostasis, inflammation, proliferation, and tissue remodeling/maturation. These stages unfold consecutively, with some degree of overlap [22]. Platelets and inflammatory cells mobilize to the wound site, releasing coagulation factors such as fibronectin. Simultaneously, blood clot formation occurs, creating a temporary barrier. This phase also triggers vascular contraction, which aids in containing blood loss and prepares the wound for subsequent healing stages [23,24].

Following the completion of the hemostasis phase, the inflammatory response initiates immediately. This pivotal stage involves a meticulous process in which foreign bodies, bacteria, and dead cells are actively cleared from the wound area. The primary objective is to minimize the risk of infection, a factor known to impede the wound healing process. By efficiently eliminating potential sources of infection, the inflammatory stage establishes a conducive environment for subsequent phases of wound healing, facilitating an optimal and timely recovery. This process is carried out by inflammatory cells in the bloodstream [25,26]. During the inflammation stage, inflammatory cells release reactive oxygen species (ROS), and ultimately, macrophages and neutrophils eliminate microorganisms and tissue debris.

In the third stage of wound healing, known as proliferation, a series of intricate processes unfold. These include the formation of granulation tissue, the establishment of new blood vessels (neovascularization), the synthesis of fibrous tissue (fibrogenesis), wound contraction, and the restoration of the epithelial layer (re-epithelialization). This dynamic phase actively promotes the structural and functional restoration of the wounded tissue, marking a crucial step towards complete recovery. New granulation tissue and extracellular matrix (ECM) are developed through epithelialization, and this phase continues until the wound is closed. The proliferation of fibroblasts is the main activity during this phase [27,28]. Although the wound is not completely repaired by the end of the proliferation phase, filling the defect areas and covering it with the epidermis layer, protects the wound surface from the invasion of foreign bacteria and viruses.

**Fig. 1:**
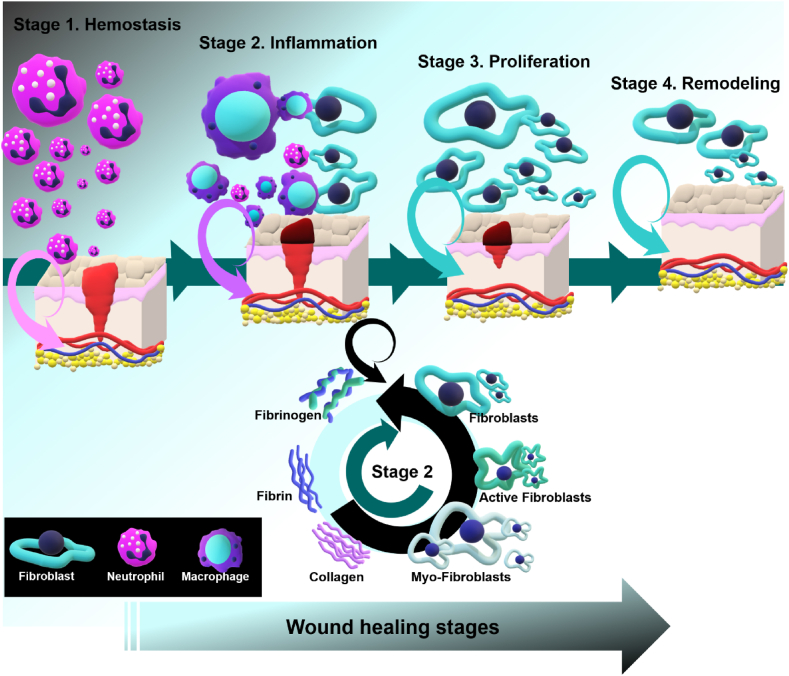
Four consecutive and overlapping phases of wound healing.

The fourth and final stage of wound healing is remodeling and maturation. During this phase, the previously temporarily repaired wound area undergoes a systematic transformation, being replaced with tissue that closely resembles the original. A specific molecular transformation occurs during the wound healing process, wherein type III collagen is replaced by type I collagen. This substitution serves to enhance the tensile strength of the newly formed tissue, contributing to its resilience and structural integrity [29,30] (see Fig. 1).

**Fig. 2:**
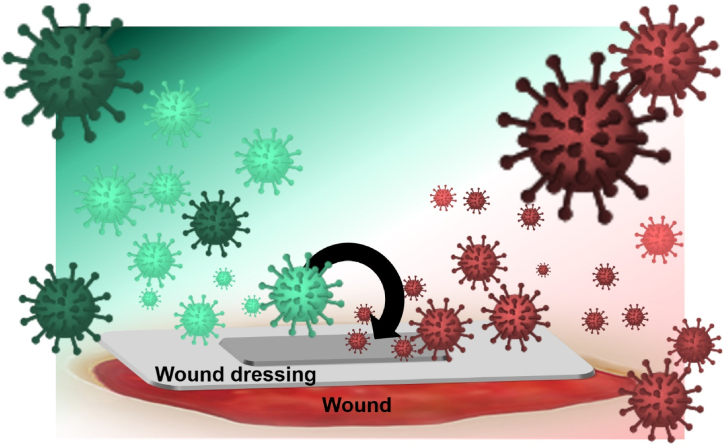
illustrates how wound dressing functions as a protective shield, separating the injured tissue from the ambient surroundings.

The critical choice of a suitable technique for wound treatment underscores its importance. Among the foremost and readily available methods is the application of dressings. Ordinarily, wound dressings play a pivotal role as a physical barrier, strategically positioned to create a protective interface between the wound and the external environment. These dressings not only shield the wound from potential contaminants but also actively contribute to maintaining an optimal healing environment by regulating moisture, temperature, and other essential factors (Fig. 2). This helps prevent additional damage or infection and expedites the process of wound healing.

Currently, depending on the type of wound, there are different types of dressings available, including dry dressings (for example, gauze, bandage or cotton wool) [31], transparent dressings [32], hydrocolloids [33], hydrogels [34], foams [35] and nanofibers [36].

**Fig. 3:**
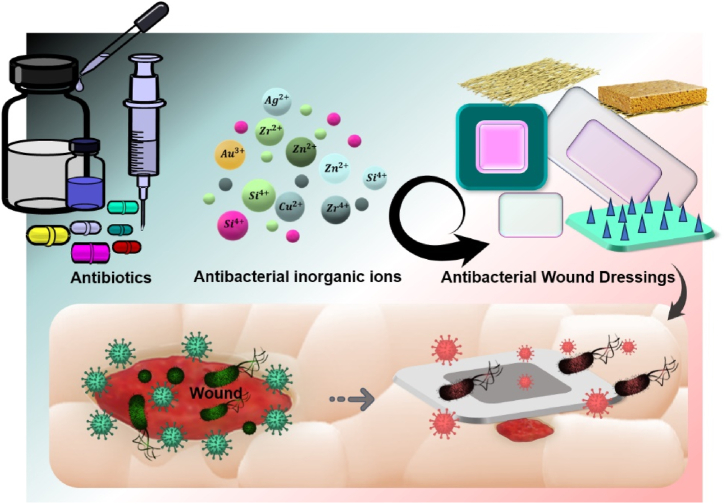
Covering the wound surface with antibacterial agent-loaded dressings prevents the penetration of microorganisms and wound infection.

Generally, an ideal bandage should possess several basic properties, including the promotion hemostasis, pain relief, sufficient mechanical properties and flexibility, and the ability to maintain wound moisture [[37], [38], [39], [40], [41], [42], [43], [44]]. Considering the hindrance microorganisms pose to the wound healing process, an ideal dressing should not only possess antibacterial activity but also exhibit efficacy in preventing infection and disrupting the formation of bacterial biofilms. This dual functionality is crucial for creating an environment that fosters optimal healing conditions by actively inhibiting microbial colonization and promoting a sterile milieu within the wound site. The efficacy of antibacterial dressing is illustrated in Fig. 3, showcasing the incorporation of agents such as drugs and bioceramic nanoparticles that effectively penetrate microorganisms, and prevent wound infection. Due to incorrect and excessive use of antibiotic drugs, bacterial resistance may occur, and the use of ceramic-based antibacterial nanoparticles is superior in this regard. These nanoparticles destroy bacteria and fungi or inhibit their growth by disrupting their natural metabolism or directly affecting the bacterial cell membrane [45,46].

Other basic features of modern wound dressings include bioactivity, stimulation of cell behavior and angiogenic activity. Many physiological processes in the body, including wound healing, are regulated by ions. The ionic products resulting from the degradation of bioceramics serve as biochemical cues can control cellular functions through intracellular signaling, thus, the metabolism of the cells is dependent on these ions [47]. A variety of essential ionic elements, including but not limited to calcium (Ca), phosphorus (P), zinc (Zn), copper (Cu), silver (Ag), magnesium (Mg), titanium (Ti), strontium (Sr), zirconium (Zr), silicon (Si), and aluminum (Al), play pivotal roles in influencing the wound healing process. In addition to hard tissue repair, the effectiveness of bioceramics in soft tissue repair has also been studied [48]. Table 1 offers a comprehensive overview, detailing the specific contributions of these ions to different aspects of the wound healing journey.

**Table 1:** Roles of metallic ions in the wound tissue regeneration and healing process.

Ion	Role in the Tissue Regeneration and Healing Process	Ref.
Ca^2+^	- Generation of thrombin and coagulation - Chemokine effects - Fibroblasts adhesion and proliferation - Keratinocytes differentiation - Controlling the activity of genes associated with angiogenesis	[50,51]
Mg^2+^	- Fibroblast cells' migration, adhesion, proliferation and differentiation - Collagen formation - Angiogenesis - Regulation of the inflammatory process	[52,53]
Zn^2+^	- An important ion in coagulation process - Cellular immune regulation - Epithelial regeneration - ECM synthesis - Antimicrobial activity	[52,54]
Sr^2+^	- Stimulation of proangiogenic factors expression - hair follicle regeneration	[55,56]
Si^4+^	- Stimulation of pro-angiogenic growth factors secretion - Synthesis of collagen - Glycosaminoglycans synthesis - Improvement of endothelial cells viability	[57,58]
Cu^2+^	- Involvement in vessel formation and angiogenesis - Inducing VEGF expression and angiogenesis - Antibacterial activity - Improvement of keratinocytes adhesion via induction of integrins expresion	[59]
Ag^+^	- Significant antibacterial activity	[60,61]
Au^3+^	- Anti-inflammatory and antimicrobial properties	[62,63]
Zr^4+^	- Antibacterial, antioxidant and anti-inflammatory activity	[64,65]

**Fig. 4:**
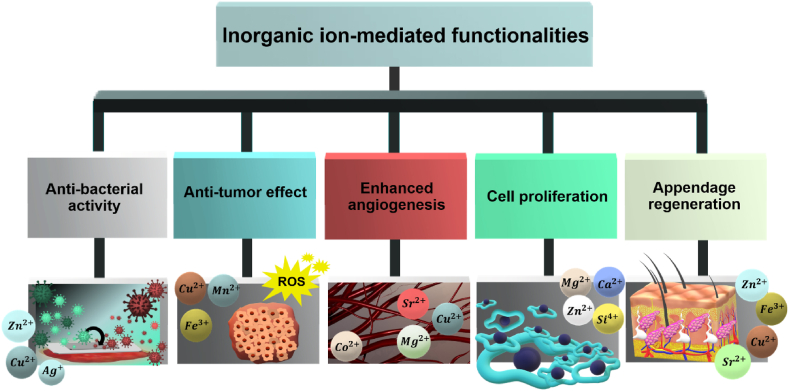
Schematic illustration of ion-mediated functionallities in skin tissue repair.

Inorganic/metallic ions naturally control epidermal barrier and enzymatic activities, maintain redox balance, and facilitate skin remodeling [49]. The inorganic/metallic ions liberated from bioceramics can additionally trigger antibacterial and antitumor activities, along with promoting angiogenesis and the regeneration of skin appendages (Fig. 4). Indeed, ionic products released from bioceramics in the biological environment, act as biochemical cues and by directly affecting cell behavior through intracellular signaling can regulate cellular functions [47].

## 3 Cost-Effectiveness of using nanobioceramics

The cost of bioactive substances such as bioceramics, growth factors or active pharmaceutical ingredients for treating wounds is a key indicator, determining whether they can be mass-produced or not. The cost of production can be considered including raw materials, time, equipment and human resources, excluding taxes and insurance premiums. Since the growth factors and most of the effective pharmaceutical substances are organic compounds and require very complex extraction and purification processes, they are usually more expensive to produce than the bioceramics with relatively affordable precursors. For instance, it has been reported that the production of some growth factors may cost up to 400,000 USD per gram [66]. Table S1 presents the price of some commercially available growth factors, active pharmaceutical ingredients, and various precursors required for the synthesis of different bioceramics. Accordingly, the most expensive precursor of bioceramics is silver nitrate with a price of about 5.8 USD per gram, while the price of each micrograms of growth factors such as VEGF and IGF-1 is around 51.15 and 6.57 USD, respectively. On the other hand, the pure active ingredient of the widely used drugs Tetracycline and Ciprofloxacin are priced at 6.40 and 6.66 USD per grams, respectively. It is worth noting that some pure pharmaceutical ingredients such as Mupirocin and Simvastatin have a much higher price (11.24 and 13.28 USD per milligrams, respectively). This means that apart from the effectiveness, the synthesis and use of bioceramics is much more cost-effective than growth factors and many pure pharmaceuticals.

## 4 Nanobioceramics in wound healing

Nanobioceramics represent a versatile category of nanoceramics employed in regenerative medicine and biomedical applications, prized for their exceptional biocompatibility and effective interaction with living tissues. Generally, nanobioceramics are classified into three categories of bioinert, resorbable and bioactive. Because of their excellent mechanical properties, bioceramics are primarily used to replace or repair hard tissues. Nevertheless, the effectiveness of inorganic ions emanating from bioceramics extends to activating soft tissue cells, such as fibroblasts, endothelial cells, and epithelial cells, while also modulating macrophage polarization. This has led to the proposition of utilizing nanobioceramics for the regeneration of soft tissues [67]. Additionally, many bioceramic nanoparticles exhibit strong antibacterial activity and unlike antibiotics, their efficacy is not affected by bacterial resistance [68,69].

**Fig. 5:**
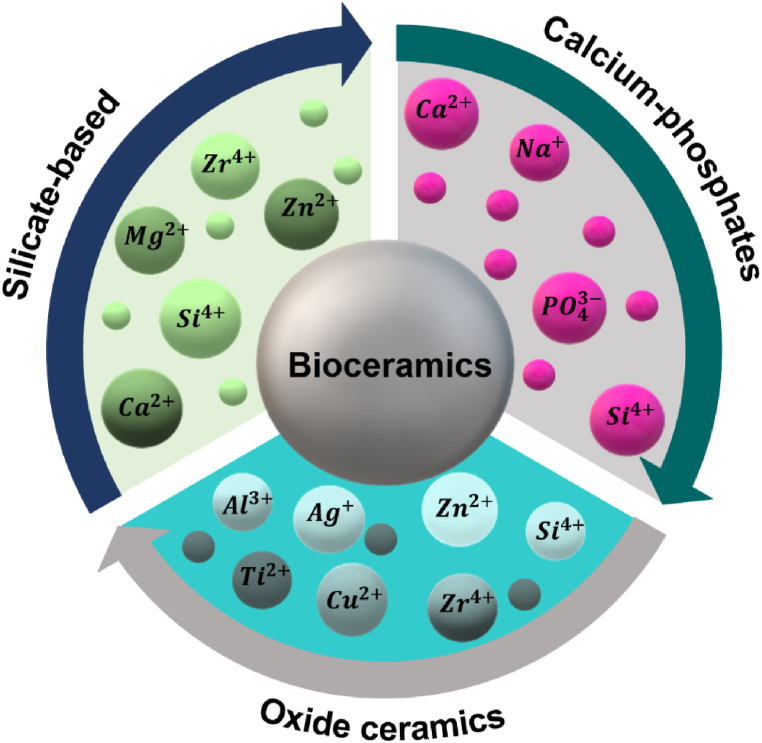
Three main categories of bioceramics and common inorganic ions in their structure.

In this study, three basic categories of bioceramics (i.e., oxide ceramics, silicate-based ceramics, and calcium-phosphate ceramics) are examined in terms of their application in wound healing (Fig. 5). These substances are often used as additives in dressings to stimulate cells and induce antibacterial and angiogenic properties. The efficacy of these materials hinges on crucial attributes, including dimensions and architecture (smaller particles exhibiting heightened biological activity), surface activation, zeta potential, and dispersion index. Various attributes, such as porosity, chemical structure, heterogeneity, and hydrolytic stability of bioceramic particles, are critical factors influencing not only their biological behavior but also their overall performance. These properties collectively contribute to the biocompatibility and effectiveness of bioceramics in diverse applications, ranging from tissue engineering to medical implants [70].

### 4.1 Oxide ceramics

Compounds comprising metallic and metalloid elements, including Al, Zr, Ti, Cu, Zn, Ag, and Si, bonded with oxygen (O), fall under the category of oxide ceramics. Renowned for their biocompatibility, resistance to corrosion, and exceptional mechanical strength, these materials stand out for their versatility across various applications, showcasing their durability and compatibility with a wide range of uses.The performance of oxide ceramics is influenced by factors such as grain sizes, crystal structures, defects, and chemical compositions. Notably, these ceramics exhibit resistance to corrosion and degradation by biological organisms, possessing molecular structures distinct from living tissues and generally maintaining stability within the body. In addition, they can mediate both adhesion and proliferation functions of different cell lines including, mesenchymal stem cells [71]. Table 2 provides a compilation of earlier research endeavors exploring the utilization of oxide ceramics in the context of wound healing.

**Table 2:** Overview of selected studies exploring the application of oxide ceramics in wound healing.

Bioceramic System	Bioceramic type	Description	Result	Ref
Oxide ceramics	Al_2_O_3_	Nanoporous AAO used for skin regeneration application	Generating elevated levels of ROS, heightened apoptosis, and impairment of keratinocyte endothelial cells, fibroblast, and collagen metabolism are observed outcomes. The AAO membranes exhibit promising attributes, including reproducibility, uniformity, biocompatibility, and cost-effectiveness, rendering them potentially suitable for clinical applications.	[88]
		Al_2_O_3_ NPs with liquid particles (immersed in deionized water) treated with laser for wound healing purpose	Wound healing activity for Al_2_O_3_ NPs showed high effect of antibacterial activity against *S. aureus*, also antioxidant activity which leads to healing in mice.	[89]
		The nanofibers were prepared by enriching Al_2_O_3_ NPs with honey/chitosan and clove extract (CE), resulting in the formation of HTCs-CE, HTCs-Al_2_O_3_ NPs, and HTCs-CE/Al2O3 NPs nanofibers	Enhanced bacterial growth inhibition efficiency was observed. The nanofibers exhibited superior biocompatibility, cell viability, and cell proliferation compared to commercially available counterparts.	[90]
		45S5 nano Bioactive Glass- Al_2_O_3_ Composites	Enhancement in apatite formation/increased cell proliferation rate, cell growth and migration.	[91]
	ZrO_2_	Implants made of ZrO_2_ and Ti were inserted into 22 patients.	The survival and success rates for zirconia implants reached 93.3 %, while Ti implants achieved a 100 % success rate over a 24-month period. All patients experienced uneventful wound healing.	[92]
		Collagen scaffolds with hybrid stabilization through oxidized inulin and ZrO_2_ nanoparticles.	Scaffolds, compatible with both stem cells and osteoblast cells, exhibit a wound healing capacity of up to 60 % following 24 h of incubation. The greater cell migration index compared to the native collagen scaffold contributes to this remarkable effectiveness.	[93]
		Cirsium setidens extract (CSE) facilitated the loading of antibiotics (ampicillin (Amp) and erythromycin (Ery)) onto ZrO_2_ NPs.	No evidence of cytotoxicity observed in NIH3T3 cells, chick chorioallantoic membrane (CAM), and red blood cells (RBC). Healing efficiency of ZrO_2_-Amp NPs and ZrO_2_-Ery NPs, although lower than ZrO_2_ NPs, surpassed untreated control and antibiotic groups. Remarkably, ZrO_2_-Amp NPs and ZrO_2_-Ery NPs retained the ability to stimulate cell growth post-antibiotic loading.	[94]
	TiO_2_	Patch made of PVA/SA/TiO_2_-CUR	The PVA/SA/TiO_2_-CUR patch exhibited significant capabilities in swelling. Demonstrated efficient drug release from the patch. Controlled evaporation rate observed. Positive interaction with blood. High cell viability indicated. Antibacterial activity validated. Effective wound healing demonstrated. Overall suitability established for addressing epidermal damage.	[95]
		Diamond-like carbon (DLC) films containing TiO_2_ nanoparticles	Peritoneal macrophages of mice in contact with DLC and TiO_2_-DLC implants did not exhibit any signs of infection	[96]
		A hydrogel comprising PVA with incorporated Ag/TiO_2_.	Enhanced wound healing activity attributed to TiO2 NPs. Astringent and antimicrobial properties of TiO_2_ NPs contribute to enhanced healing. Increased cell adhesion observed. Improved photocatalytic antibacterial activities against both Gram-positive and Gram-negative bacteria. Significant acceleration in wound healing in a rat model compared to traditional dressing methods.	[97]
		A bio-nanocomposite film incorporating TNTs and Gellan Gum (GG).	Inclusion of TiO2-NTs enhances mechanical properties. Higher concentrations of TiO^2^-NTs correlate with reduced water uptake and swelling. Increased tensile modulus observed with higher TiO_2_-NT concentrations. Nanocomposite-treated wounds display significantly accelerated healing. Suppression and diminishment of the inflammatory response noted. Activation of re-epithelialization observed in wounds treated with the films.	[98]
		A three-dimensional nanocomposite scaffold incorporating TNTs and carrageenan.	Stimulate cell viability and proliferation and expedite the healing process. The cell count surpassed 190,000 after 3 days, contributing to an accelerated wound closure rate.	[99]
	CuO	Hydrogels with bioactive carboxymethyl Starch and CuO NPs decoration	Enhanced antibacterial properties, and antioxidant activity, as well as swelling capacity were observed. The material demonstrated cytocompatibility towards HDFs and displayed commendable biocompatibility.	[100]
		A hydrogel spray with adhesive properties loaded with hybrid hollow mesoporous CuO nanospheres with virus-like characteristics containing glucose oxidase.	Catalyzes intracellular H_2_O_2_ to generate abundant O_2_. Alleviation of hypoxia demonstrated. Effective enhancement observed in the healing of diabetic wounds. Serves as a reliable tool for chronic non-healing wounds.	[101]
		Wound dressings with antibacterial properties, infused with COD, for clinical investigation.	Novel wound healing approach leveraging the capacity of Cu^2+^. Promotion of angiogenesis and epithelialization properties demonstrated.	[102]
	SiO_2_	Utilized copper peroxide nanodots in a spray form to expedite the healing process of a diabetic ulcer infected with multi-drug-resistant bacteria.	Enhanced cell migration and tube formation observed. High expression of HIF-1α and VEGF in HUVECs. Effective antibacterial properties noted. Induced angiogenesis observed. Accelerated wound healing *in vivo* for MRSA-infected diabetic ulcers.	[103]
		Treatment involving SiO_2_/Au nanoshells and a diode lase	The tensile strength rose with higher concentrations of both gold nanoshells and SiO_2_. Given that the skin temperature should not surpass approximately 90 °C, these conditions were deemed acceptable for our experiment, making them ideal for wound enclosure.	[104]
		Bilayers assembled from poly(allylamine hydrochloride) and SiO_2_ mesoporous nanoparticles for humidity sensing within the skin microenvironment.	Settling time exceeded that of the commercial sensor. Efficient wound healing is supported by flexibility, ease of integration into dressings, and immunity to electromagnetic fields.	[105]
		Catheter coated with porous Se/SiO_2_ nanospheres.	Reducing the inflammatory response, It shifted macrophage polarization towards the M_2_ phenotype by inhibiting the ROS-NF-κB pathway.	[106]
		Nano composites of Curcumin/Fe-SiO_2_	Collaborative action in controlling scar and hair follicle formation. Substantially impeded scar hyperplasia and fostered hair follicle regeneration.	[107]
	ZnO	Wound dressings crafted from cotton containing Ag and ZnO NPs, along with a composite blend of Ag and Ag/ZnO NPs.	Bandages immersed in a liquid solution of Ag NPs exhibited superior antimicrobial effectiveness compared to those treated with ZnO NPs and Ag/ZnO NPs.	[108]
		Bacterial Cellulose- ZnO NPs	Incorporating significant antimicrobial efficacy into bacterial cellulose demonstrated improved wound healing and tissue regeneration capabilities.	[109]
		Hydrogel composed of Ch and PVA, infused with heparinized ZnO NPs	The concentration required for inhibition decreased upon the surface conjugation of heparin with ZnO NPs. The addition of heparin to nanoparticles resulted in an increased antibacterial synergistic effect. This promoted rapid wound healing compared to the control sample, enhancing re-epithelialization and collagen formation.	[110]
		Biodegradable thiolated bandage embedded with ZnO-NPs for preventing postoperative surgical site infections.	Notable enhancement in tissue regeneration was observed, with favorable properties such as lysozyme degradation and porosity conducive to wound healing. The *in-vitro* bactericidal effect exceeded that observed in bandages lacking ZnO-NPs.	[111]
	Ag_2_O	Nanocomposite Modification of Cellulose Acetate Films with Ag_2_O and ZnS	Effective antibacterial performance against *E. coli*, Excellent thermal stability and cell viability, Uniform dispersion of additives in cellulose acetate	[112]
		Injectable Hydrogel of Methylcellulose with Ag_2_O NPs	Outstanding antimicrobial efficacy and burn wound healing, Exceptional epithelial restoration, Accelerated gelation time	[113]
		Ch-PVP-nano Ag_2_O NPs	Good swelling capability, low cell toxicity and high cell viability (L929), Quick re-epithelization of epidermis and wound healing	[22]

*Aluminum oxide (Al*_*2*_*O*_*3*_*);* The only oxide formed by aluminum in nature is aluminum oxide (Alumina, Al_2_O_3_). Aluminum occurs naturally in various forms, including corundum (Al_2_O_3_), diaspore (Al_2_O_3_·H_2_O), gibbsite (Al_2_O_3_·3H_2_O), and bauxite, which is an impure variation of gibbsite. Functioning as an active amphoteric metal, aluminum readily develops a white oxide film under normal conditions. Al_2_O_3_ is best known for its phase modifications α-, β-, and γ- Al_2_O_3_ [71]. The tensile strength of *Al*_*2*_*O*_*3*_ metal matrix composites increased with decreasing particle size and increasing particle number. Composites reinforced with 10 wt% Al_2_O_3_ particles exhibit higher tensile strength, in comparison with other oxide ceramics. Alumina reveals outstanding features, particularly in terms of friction and wear resistance, with the most notable effects observed when the grains are around 4 μm, displaying a narrow size distribution [72]. Topics related to wound healing, including infection, have been highly emphasized. It is important for public health to find new ways to control antibiotic-resistant bacterial infections. In eukaryotic animal cells, Al_2_O_3_ NPs demonstrate low toxicity without inducing apoptosis. Nevertheless, studies indicate that Al_2_O_3_ can induce oxidative stress and neurotoxicity, potentially leading to neuropathologies [73]. Al_2_O_3_ NPs have shown significant effectiveness in applications related to wound healing, supported by compelling evidence from both *in-vitro* and *in-vivo* studies. Further specifics can be referenced in Table 2. Al_2_O_3_ has not been extensively used in clinical studies for wound healing, especially among diabetic patients.

*Zirconium oxide (ZrO*_*2*_*);* Zirconia (ZrO_2_) displays three distinct polymorphs: monoclinic, tetragonal, and cubic. Research findings indicate that a ZrO_2_ coating with lateral spacing adopts a structure resembling the ECM, leading to improved adhesion and proliferation of L-929 cells [73]. Moreover, composites containing ZrO_2_ have shown the capacity to prompt the differentiation of mesenchymal stem cells [74]. ZrO_2_ has undergone evaluation for wound healing applications through randomized clinical trials, as well as *in-vitro* and *in-vivo* studies. The findings of these investigations are summarized in Table 2.

*Titanium dioxide (TiO*_*2*_*);* Different nanostructures of TiO_2_, including nanorods, nanotubes, nanowires, and nanobelts, have been synthesized through nanotechnology studies. The production of TiO_2_ nanostructures commonly employs methods such as template-assisted, electrochemical, and hydrothermal processes [75]. Gulati et al. [76] investigated TiO_2_ nanotubes (TNTs) for their topographical impact on MC3T3-E1 mouse preosteoblasts. The TNTs exhibited a decrease in both Ras homolog family member A (RhoA) and focal adhesion kinase (FAK), impacting actin stress fibers, actomyosin contractility, and ECM/integrin-regulated proliferation. Additionally, the investigation revealed remarkable enhancements in cell adhesion and proliferation in pores with a diameter of 30 nm or less, in contrast to minimal cellular responses in pores larger than 90 nm. Despite the *in-vitro* studies on TiO_2_ nanostructures, there is a critical need for increased attention to their application in clinical fields. More research related to these bioceramics can be found in Table 2.

*Copper oxide (CuO);* The versatile capabilities of Cu and CuO NPs position them as ideal candidates for various applications, including antimicrobial, catalytic, anticancer, photocatalytic, antiviral, biofilm synthesis, nitrate removal, anti-human pathogens, and photoluminescent activities, along with catalyzing the degradation of organic dyes. Cu plays a crucial role in fostering angiogenesis and facilitating the synthesis and stabilization of ECM proteins in the skin. These processes are essential for the inherent healing of skin tissue. Borkow et al. [77] utilized a wound dressing infused with copper oxide (CuO) to address wounds in diabetic mice. The outcome revealed upregulated gene expression and in situ presence of proangiogenic factors, such as placental growth factor, hypoxia-inducible factor-1 alpha, and vascular endothelial growth factor, which results in improved blood vessel formation. Furthermore, CuO shows its capability to directly facilitate the healing of non-infected, challenging wounds on the feet of diabetic patients [78]. Further details on studies related to the wound healing applications of CuO can be found in Table 2.

*Silicon oxide (SiO*_*2*_*);* SiO_2_ NPs are extensively employed synthetic materials, finding widespread use in numerous *in vitro* and *in vivo* applications. To reach its dressing application, other polymers like PVP has been mixed in with them and cross-linked to form the dressing [71,79]. Mesoporous materials have emerged as promising candidates for delivering various drug molecules in a controlled and sustainable manner. Among these, mesoporous SiO_2_ NPs are widely utilized as delivery agents due to the favorable chemical properties, thermal stability, and biocompatibility inherent in silica. These materials feature ordered arrangements of pores ranging from 2 to 50 nm, creating channels and cavities of diverse shapes. Notably, the 2D hexagonal planar MCM-41 and SBA-15 structures (symmetry group p6mm) with approximately 2 and 10 nm pores, respectively, and the 3D-cubic MCM-48 (symmetry group la3d) with around 3 nm pores are common ordered mesoporous frameworks within SiO_2_ [80]. *In vitro* toxicology studies have indicated the tolerability of mesoporous SiO2 NPs (MSNs) by mice, even at dosages below 100 g/mL. In conventional MSNs, one therapeutic drug dose is loaded per 1 gr of SiO_2_, typically ranging from 200 to 300 mg, with a maximum of 600 mg [81]. Li et al. [82] demonstrated the proliferation-promoting effect of 500 nm-SiO_2_@AuNPs on NIH/3T3. Therefore, SiO_2_–Au core–shell materials can treat cutaneous wounds. Table 2 shows the wound healing studies using SiO_2_.

*Zinc oxide (ZnO);* Zinc oxide (ZnO) typically crystallizes in two primary forms: hexagonal wurtzite and cubic zinc blende. ZnO has experienced a substantial increase in biomedical applications, spanning drug delivery, bioimaging, gene delivery, biosensors, and wound healing processes. ZnO NPs, with their antibacterial properties, emerge as intelligent agents against multiple drug-resistant microorganism and serve as a promising alternative to antibiotics [83]. Furthermore, ZnO is utilized in dermatological products for its effectiveness in reducing inflammation and itching, and also accelerating the wound healing process. In dentistry, it is employed for temporary fillings and dental pastes. Due to its capability to deliver essential dietary Zn, ZnO is incorporated into various nutritional products and dietary supplements. At the nanoscale, ZnO serves as a UV radiation blocker, catalyst, and biomaterial in the biomedical field, contributing to diagnosis and treatment [84]. ZnO NPs of larger particle sizes exhibit a high viability rate for fibroblast cells. The antimicrobial activity of ZnO NPs is contingent on particle size, with smaller particles demonstrating enhanced efficacy in combating various microorganisms [85], as highlighted in Table 2, where its applications in wound healing are detailed.

*Sliver Oxide (Ag*_*2*_*O);* Ag_2_O NPs display promising potential in wound treatment, offering a viable means to reduce the risk of limb amputation. Known for their outstanding properties, such as remarkable antibacterial activity, an effective anti-inflammatory response, and non-toxic characteristics, these nanomaterials emerge as an optimal choice for wound dressings, ensuring effective and safe applications in wound care [86]. In addition to their antimicrobial efficacy, Shakya et al. [87] highlight the appropriate physicochemical properties of Ag_2_O nanoparticles, which enable surface functionalization through the coordination of specific ligands. While researchers have demonstrated the biocompatibility of Ag_2_O NPs for wound healing, exploration for commercial products has been limited to only a few compounds based on Ag_2_O. Table 2 provides a comprehensive overview of the application of these nanoparticles in prior studies.

### 4.2 Silicate-based ceramics

Lately, there has been growing interest in biodegradable silicate-based bioceramics, especially within the realms of bone tissue engineering and applications related to wound healing. The inclusion of ions like Ca, Mg, and Si in silicate bioceramics is recognized for its significant impact on cell proliferation, differentiation, and growth, as well as angiogenesis, alleviation of inflammatory reactions, antibacterial effectiveness, and support for re-epithelialization under physiological conditions at the wound site [[114], [115], [116]]. Silicate-based bioceramics that have been utilized for wound healing include Akermanite (Ca_2_MgSi_2_O_7_), Diopside (CaMgSi_2_O_6_), Calcium sodium silicate (Na_2_CaSiO_4_), Wollastonite (CaSiO_3_), Zeolites (M_2/_nOAl_2_O_3_.γSiO_2_.wH_2_O), Bredigite (Ca_7_MgSi_4_O_16_) and Hardystonite (Ca_2_ZnSi_2_O_7_).

A recent study [117] proposed that despite akermanite's widespread use as a calcium silicate-based ceramic in bone regeneration, it exhibits potential applications in wound healing either independently or as part of a composite material. Furthermore, akermanite powder impact on wound healing was explored *in vivo*. Remarkably, wounds treated with akermanite powder exhibited complete healing after 14 days compared to the control group. Akermanite promoted re-epithelialization by triggering the Wnt/β-catenin pathway, thereby enhancing the migration and stemness of epidermal cells at the wound site.

Additionally, *in vitro* assessment of akermanite ion extracts at a 1/16 optimum ratio (diluted with EpiLife™ medium) revealed enhanced cellular viability, increased proliferation, improved migration ability, and heightened stemness of human epidermal stem cells (hESCs), possibly attributed to the high-expressing EGF/EGFR/ERK signaling pathway. The observed positive effects of akermanite on hESCs' biological functions may be linked to the release of silicon within the optimal range of 0.7–1.8 μg/mL for concentration. In a relevant investigation [118] researchers fabricated a composite hydrogel containing injectable bioactive akermanite and alginate by internal gelation method. In this study, Ca^2+^, Mg^2+^ and Si^2+^ dual ions released from akermanite act as crosslinking agents of alginate chains with the assistant of acidic amino acids, which increased compressive strength of the composite hydrogel (150 kPa) compared to the hydrogels crosslinked with single Ca^2+^ (50 kPa). In addition, the *in-vivo* evaluations of employed composite hydrogel showed that the bioactive ions released from akermanite associated with the porous and hydrated structure of hydrogels could stimulate cell migration followed by angiogenesis, the epidermis formation and collagen deposition in the wound site, and finally accelerate the wound healing.

In another study [119] it was noted that integrating akermanite nanoparticles into a wound dressing composed of tragacanthin gum-carboxymethyl chitosan bio-nanocomposite has the potential to enhance porosity, leading to decreased scaffold density and swelling. The enhanced penetration of oxygen and proteins to the wound site was facilitated. Furthermore, the introduction of calcium silicate nanoparticles not only strengthened the polymer's mechanical properties but also concurrently intensified the surface interaction between the polymer and nanoparticles.

The presence of microbial infections can significantly prevent the wound healing process and worsen the severity of wounds. Hence, the antibacterial properties of calcium silicates in wound dressings need to be considered. In a particular study, the antibacterial activity of diopside, akermanite, and merwinite bioceramics was compared at various concentration levels (500 μg/100 μL, 750 μg/100 μL, and 1 mg/100 μL) against model bacteria including *Staphylococcus epidermidis*, *S. aureus*, *E. coli*, and *Pseudomonas aeruginosa* [120]. Merwinite exhibits significant antibacterial efficacy across all bacterial species, even at lower concentrations (500 μg/100 μL), better than diopside and akermanite. Notably, akermanite's antibacterial activity was limited to *S. epidermidis* at higher concentrations due to its low solubility in aqueous media. Overall, the antibacterial properties of silicate ceramics are attributed to the elevated aqueous pH resulting from the exchange of Ca^2+^ ions with protons from the aqueous medium.

Another investigation [121] found that employing calcium silicate (Na_2_CaSiO_4_) effectively enhanced the viability, growth, proliferation, and migration, as well as fibroblasts differentiation induced by high glucose. This, in turn, facilitated the tissue regeneration and healing of diabetic wounds. The silicon ions extracted from calcium silicate also alleviated fibroblast senescence induced by high glucose by suppressing the generation of ROS, known contributors to cellular aging in diabetes. The potential mechanisms underlying the improved fibroblast functions involved higher Smad2 phosphorylation which was accompanied by enhance of fibroblast differentiation into myofibroblasts, and reduced expression of markers of cellular senescence including p53, p16, and p21. Additionally, the *in-vivo* experiment on diabetic mice was observed to enhance collagen deposition and myofibroblast differentiation.

Metal ions have demonstrated a promising potential to react with silica ions present in calcium silicate ceramics, leading to the development of innovative characteristics. In a study conducted by Abudhahir et al. [122] the cytocompatibility of Zn-doped Wollastonite (Zn-Ws) nanoparticles was assessed using FDA and DAPI staining assays on Fibroblast cells (NIH3T3). The presence of Zn-Ws particles prompted cell spreading, with no observable morphological changes, thereby affirming favorable cytocompatibility within a specific concentration range of 0.2 mg/mL. Moreover, an increase in protein adsorption occurred more rapidly in Zn-Ws particles as compared to Ws particles over time. This ultimately leads to an acceleration of fibroblast proliferation toward wound healing. This occurrence might be due to the increased surface area stemming from the interaction of Zn^2+^ (tetrahedral Zn(O, OH)_4_) with silicate anions.

In a distinct investigation [123] calcium silicates were utilized as in situ cross-linking agents and bioactive elements in creating hydrogels with manganese-doped calcium silicate nanowires. Metal ions like Mn^2+^ can chelate with a sodium alginate solution, forming a network structure gel. However, the rapid reaction rate hinders the formation of uniform hydrogels. The presence of Ca^2+^ ions in calcium silicates limits manganese release during gelation due to the manganese doping into calcium silicate nanowires and allows Mn^2+^ to occupy silicate vacancies or replace Ca^2+^, resulting in a higher release of Ca^2+^ than Mn^2+^ during gelation. Additionally, assessments conducted both *in vitro* and *in vivo* demonstrated the collaborative impact of Si and Mn ions in improving the wound healing process. When applied to diabetic mice, hydrogels comprising nanowires of calcium silicate doped with manganese facilitated faster healing, exhibiting higher densities of blood vessels, hair follicles, glands, and other skin appendages compared to calcium alginate and calcium silicate alginate after 15 days, as revealed by histological results.

Bredigite (BR) belongs to a magnesium silicate based bioceramic and exhibits an remarkable ability to produce apatite similar to that found in bone in simulated bodily fluids and proper wound healing with biocompatibility. An investigation assessed the impact of introducing BR NPs into fibrinogen (FG)/Poly (3-hydroxybutyrate-co-3-hydroxyvalerate) (PHBV)/BR nanofibrous membranes. Findings indicated that the inclusion of BR elevated the nanofiber structure with nanofiber diameter to 315 ± 50 nm, resulting in larger membrane pore diameter conducive to enhanced diffusion of oxygen, fluids, and nutrients. Additionally, the incorporation of BR NPs enhanced hydrophilicity, potentially promoting improved cell adhesion and proliferation [124].

Hardystonite (ZnCS) stands out as a well-known calcium silicate ceramic utilized in conjunction with wound dressings to enhance the efficacy of wound tissue regeneration. Explored in a reserch [125] the effectiveness of a layer containing hardystonite particles combined with hydrophobic polylactic acid (PLA) in healing burn wounds and regenerating hair follicles in a rat burn model was studied. In this research, hydrophilic hardystonite powders were strategically placed between hydrophobic PLA fibrous sheets through hot pressing. The findings indicated that the resulting Janus membrane, created by combining the ceramic powder layer and the hydrophobic PLA layer, possessed characteristics suitable to effective exudate transportation from the wound bed to the dressing. This emphasized a fluid transfer from the polymer side to the ceramic side. Conversely, the hydrophobic PLA fiber membrane, positioned as the bottom layer in this design, served as a preventive barrier against the direct adherence of hydrophilic bioceramic powder to the wound. Furthermore, the integration of dressing with ZnCS bioceramic particles was observed to enhance various aspects in a third-degree burn model. These enhancements included hair follicle dermal papilla cells (HHDPCs), epithelial regeneration, cell migration, viability, and stem cell indicator markers expression (such as KGF, HGF, VEGF,BMP-6, etc,.). The improvement was credited to the synergistic activity of released ions, such as Zn^2+^ and SiO_3_, surpassing the effects of Zn^2+^ or SiO_3_^2−^ alone.

In a similar study [126], hardystonite was used to induce a synergistic effect in combination with polycaprolactone (PCL) and tetrafluoroethylene (TPE) in an electrospun PCL/ZnCS/TPE composite fibrous membrane. The resulting scaffolds not only stimulated cell migration, increased cell viability, and promoted cell differentiation, but the presence of hardystonite also resulted in fiber diameter change. The higher electrical conductivity in hardystonite, attributed to ions like Ca^2+^ and Zn^2+^, plays a pivotal role in this phenomenon. Increased electrical conductivity contributes to heightened tensile forces exerted on the fibers, consequently leading to a decrease in the diameter of the fibers. This phenomenon is indicative of the complex relationship between electrical conductivity and mechanical properties in fiber structures. Conversely, an increase in hardystonite content raises the solution viscosity, leading to an expansion in fiber diameter. Breifly, the use of near-field electrostatic spinning 3D printing technology in producing composite fibrous membranes results in enhanced oxygen exchange with the external environment in comparison to cast membranes, ultimately promoting improved wound healing.

In a correlated study [127], a dual ion-crosslinked injectable composite hydrogel consisting of hardystonite and sodium alginate was synthesized and its *in-vitro* biological properties was revelead using both human cells of umbilical vein endothelial cells (HUVECs) and dermal fibroblasts (HDFs), isolated from the vein of the human umbilical cord and the outer layer of mature human skin, respectively. Their results showed that divalent ions like Ca^2+^ and Zn^2+^ served as crosslinkers during the initiation and progression of gelation in the composite hydrogel. Additionally, increasing ZnCS content to 2 wt% resulted in enhanced compressive strength and gelling time. Nevertheless, above 0.5 wt%, there was no notable distinction in compressive strength and the time required for gel formation. Furthermore, *in-vitro* bioactivity assessments demonstrated that ZnCS-containing samples stimulated the viability, growth, and migration of both HUVECs and HDFs when compared to extracts from pure SA hydrogel. Moreover, the released Zn^2+^ ions from ZnCS exhibited antibacterial activity, achieving 100 % efficacy in both extract and direct contact assays.

Researchers favor zeolites in wound dressings because of their outstanding properties as a crystalline aluminosilicate microporous material. These include antimicrobial, anti-inflammatory, hemostatic, infection protection, cell penetration properties, along with an adjustable pore structure, diverse composition, and high hydrophilicity [[128], [129], [130]]. One of the other properties of zeolites is ion exchange capacity. In a study [131] gelatin/Cu exchanged faujasite (CAF) composite scaffolds were fabricated by lyophilization. In this study Cu exchanged faujasite (CAF) from a family of zeolites was chosen as a suitable antibacterial agent. The obtained scaffolds with 0.5 % (w/w) of CAF had pore sizes in the range of 10–350 m. Upon application of the scaffold in the saturating solution, the zeolite's copper ions undergo a process of exchange with monovalent or divalent ions existing in the wound, such as sodium ions.The copper ions, carrying a positive charge, subsequently adhere to the negatively charged membrane of the bacterial cell. This results in damage to genetic material through the generation of ROS. Additionally, the incorporation of CAF notably influenced the scaffold's highly porous architecture, enhancing oxygen supply and consequently promoting the viability of NIH 3T3 fibroblast cells. Some other studies about the use of silicate-based bioceramics in wound healing are given in Table 3.

**Table 3:** Overview of selected studies exploring the application of silicate-based ceramics in wound healing.

Bioceramic System	Bioceramic type	Description	Result	Ref
Silicate-based bioceramics	Akermanite	Investigation the effects of Akermanite extract on wound healing	Akermanite enhanced the expressions of markers of epidermal stem cells such as integrinβ1, Lgr4, Lgr5, and Lgr6 and activated Wnt/β-catenin signaling pathway in epidermal cells	[132]
	Calcium silicate	Calcium silicate-stimulated adipose-derived stem cells (ADSCs)	Calcium silicate enhanced proliferation of ADSCs; enhanced expression of CXCR4 from ADSCs, extracts induced migration and reduced oxidative stress of ADSCs and promote migration and angiogenic capacity of the human umbilical vein endothelial cells (HUVEC)	[133]
	Zeolite	Starch-based nanocomposite hydrogel scaffolds reinforced by zeolite nanoparticles	The inclusion of zeolite increased the transparency of scaffolds; induced antibacterial and antioxidant properties; increased epithelialization, collagen formation, angiogenesis, and inflammation reduction	[134]
		chitosan/zeolite composite films	Zeolites induced ion exchange capacity; reduced water vapor permeation	[135]
		Polylactic acid fibers loaded with nitric oxide (NO)-zeolite	The utilization of zeolite showed to augment the quantity of nitric oxide storage while diminishing the rate of gas release	[136]
		Gelatin/zeolite porous scaffold	The inclusion of zeolites within the gelatin matrix resulted in a notable increase in both glass transition temperature (37 °C) and dynamic compression modulus (∼ 737 kPa); demonstrated a controlled uptake of water and biodegradation rate when compared to the control.	[137]
		Investigating the Effect of NO-zeolite on the skin	Zeolite was utilized as NO reservoir and can improve the dramatic pro-inflammatory effect compared to nitric oxide	[138]
	Hardystonite	PLA- hardystonite Janus membrane were prepared	In addition to excellent exudate absorption, hair follicle regeneration and wound healing were promoted due to the ability to release Zn^2+^ and SiO_3_^2−^ ions.	[125]
		PCL-hardystonite nanofibers were prepared	Excellent *in vitro* cell proliferation and adhesion occurred on the nanofibers and and *in vivo* wound healing was occurred in 21 days.	[126]
	Zn doped wollastonite	Effect of Zn doped wollastonite nanoparticles on wound healing was evaluated *in vitro*	The promotion of cell adhesion and proliferation of fibroblast cells (NIH3T3) was achieved, and the synthesized nanoparticles caused adequate antibacterial activity against gram positive and negative bacteria.	[122]

### 4.3 Calcium-phosphates

Extensive research has been conducted on calcium-phosphates, a category of biocompatible and bioactive materials, for potential use in the field of bone tissue engineering. These materials possess excellent osteoconductive properties, which means they can support the growth and regeneration of bone tissue. In addition to their use in bone regeneration, calcium phosphates have also shown potential as wound dressings [139,140].

Among calcium phosphate bioceramics, Bio-glasses (BG) have been mainly used in wound healing, particularly in the form of BG particle-reinforced polymer fibers, because of their unique characteristics in promoting angiogenesis, eliciting immunogenic responses, and addressing bacterial infections, which are mainly induced by the release of various therapeutic ions [141]. Mersilk® and Mirragen® are two commercial products of BG fibrous dressings that are available at the market [142]. Furthermore, numerous studies have employed bioglasses in wound healing applications.

Utilizing cell sheet technology, a research study [143] engineered skin grafts that incorporated fibroblasts and the ionic dissolution products of bioactive glass (BG) in a carefully determined dilution ratio. The findings revealed that these BG ionic dissolution products exhibit the capacity to stimulate fibroblast migration while also increasing the secretion of vital growth factors essential for wound healing, such as VEGF, basic fibroblast growth factor (bFGF), and epidermal growth factor (EGF). Furthermore, BG was observed to foster the synthesis of key proteins like collagen and fibronectin within the ECM of fibroblast cell sheets, thereby contributing significantly to the enhancement of wound healing. The assessment, both *in vivo* and *in vitro*, of α-smooth muscle actin (α-SMA) showed fibroblast differentiation into myofibroblasts, a process that promotes wound contraction, thus expediting the overall wound healing progression.

Another study [144] revealed that a 1/128 dilution of BG extract effectively modulates the inflammatory phase during the initial phases of the wound healing process.The evidence from *in-vivo* experiments indicates that BG can decrease the inflammation response and stimulate the macrophage phenotype to switch to M_2_ macrophages **(**Alternatively activated) in the wound sites. Due to their presence, reparative cells were attracted, leading to a reduction in macrophages, which can impede the wound healing process.

In a comprehensive comparative study [145], the assessment of borate bioactive glass micro-fibers (bBG MFs) against 45S5 Bioglass® (SiG) micro-fibers revealed a pronounced superiority in the *in-vitro* bioactivity of the bBG micro-fibers. The results indicated a significant improvement in bioactive response, underscoring the potential of bBG MFs for advanced applications in biomaterials and tissue engineering. The superior *in-vitro* bioactivity of bBG micro-fibers is indicative of their enhanced ability to promote cellular interactions, mineralization, and overall biofunctional performance, making them promising candidates for innovative biomedical applications. The presence of a HA layer was proposed as responsible for attracting cells essential for wound healing around it. During a 14-day immersion in PBS, it was observed that BG micro-fibers exhibited a higher rate of apatite formation compared to SiG micro-fibers. This phenomenon was attributed to the higher biodegradation of BG micro-fibers. Furthermore, the increased Zeta-potential resulting from HA production has the potential to enhance the absorption of collagen, fibrin, and fibronectin, which effectively stabilizes the wound.

**Fig. 6:**
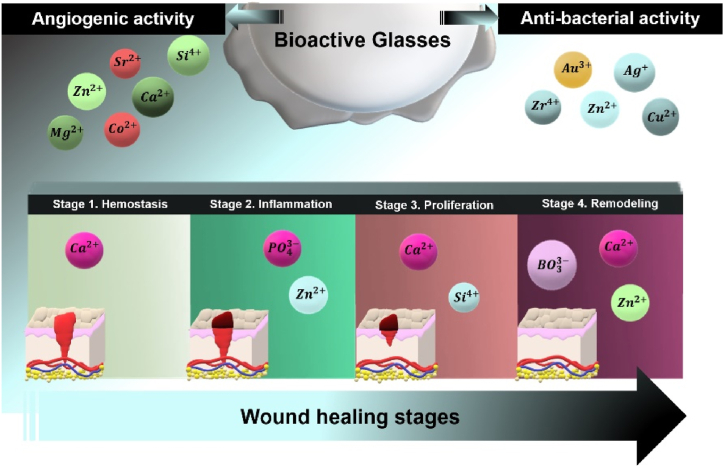
A schematic illustration outlining the contribution of various ions (both doped and non-doped) released from bioactive glasses at different stages of wound healing.

Alginate, a biocompatible material capable of forming gels with Ca^2+^, has undergone extensive research for its potential use in wound dressings [146]. In a study [147] a hydrogel composed of alginate and chitosan, enriched with Zn-doped bioactive glass (Gel-ZBG), was specifically formulated for the purpose of skin wound closure in SD rats. This innovative hydrogel aims to provide an advanced solution for effective wound healing by using the synergistic properties of alginate, chitosan, and Gel-ZBG. After 12 days of treatment, *in-vivo* investigations revealed that Gel-ZBG exhibited greater anti-inflammatory properties and exceeded the Gel (control sample) in wound closure at the treatment site. Moreover, the Gel-ZBG-EGF group showed new granulation tissue and numerous fibroblasts compared to the control sample after 6 days. These findings suggest that the system provides Si^4+^ and Ca^2+^ ions, which stimulate the secretion of advantageous factors for angiogenesis by fibroblasts and facilitate wound closure. Fig. 6 illustrates the role of different ions released from bioactive glasses in various stages of wound healing.

Presenting promising potential for wound healing applications, HA stands out due to its biocompatibility, antibacterial properties, bioactivity, and great facilitation of angiogenesis [148]. However, the inherent hardness of HA poses limitations in wound dressing applications. To overcome this challenge, researchers innovatively engineered a flexible ultralong HA nanowires-based biopaper reinforced with a small proportion of cellulose fibers (HAPNW/CF biopaper) using a vacuum-assisted filtration method. The results demonstrate that the HAPNW/CF biopaper, characterized by its high porosity, super hydrophilicity, and substantial specific surface area (36.84 m^2^ g^−1^), not only enhances cell migration, protein adhesion, and blood vessel formation but also exhibits remarkable biocompatibility. In rat models subjected to conditions of ischemia and hypoxia, the expression of angiogenesis-related factors, including endothelial nitric oxide synthase (eNOS), AKT, and vascular endothelial growth factor-A (VEGF-A) proteins, was significantly elevated in the HAPNW/CF biopaper compared to control groups [149]. This highlights the potential of the HAPNW/CF biopaper as a versatile and effective material for advanced wound healing interventions.

A crucial aspect of wound healing involves delivering antimicrobial agents to prevent infections. Local antibiotic delivery has been explored using calcium phosphate coatings and wound dressings. Despite the favorable features of HA, such as biocompatibility, proper adsorption capacity, excellent osteoconductivity, bioactivity, high surface area, and desirable mechanical strength, its application as a carrier is hindered by low drug affinity, resulting in an initial burst release upon administration. In a study, nano-HA loaded with tetracycline was encapsulated within chitosan, gelatin and, polyelectrolyte complex nanofibers. Results indicated that the presence of nano-HA increased the elastic modulus to 106 MPa, comparable to the elastic modulus of human skin (15–80 MPa). Additionally, the drug release rate decreased due to the dual physical barriers, which extended antibacterial therapeutic efficacy. Furthermore, the incorporation of nano-HA into PEC nanofibers enhanced thermotropic and hydration characteristics, making them highly suitable for prolonged applications in wound care. These improved properties position PEC nanofibers as an ideal choice for extended use in wound protection [150].

Whitlockite (WH), with the chemical formula Ca_18_Mg_2_(HPO_4_)_2_(PO_4_)_12_, constitutes approximately 25 % of human bone [151]. In a distinct investigation [152], researchers engineered a multifunctional hydrogel dressing incorporating methacrylate anhydride quaternized chitosan (QCSMA), Zn-doped whitlockite nanoparticles (Zn-nWH) and methacrylate anhydride dopamine (DAMA), through metal ion coordination and photo-polymerization with the catechol group of dopamine. The study results indicated that increasing the Zn-nWH concentration from 0.5 wt% to 1 wt% enhanced the tensile properties of the hydrogels up to 0.138 ± 0.028 MPa but reduced adhesion strength to 0.031 ± 0.002 MPa. Moreover, *in vivo*, the QCSMA/DAMA/Zn-nWH composite demonstrated a pronounced hemostatic effect, showing the shortest hemostatic time recorded at 129 ± 22 s and the lowest blood loss at 27 ± 5 mg. This performance sharply contrasts with the control group, where the hemostatic time was substantially longer at 571 ± 15 s, accompanied by a higher blood loss of 147 ± 31 mg. The presence of Ca^2+^ in WH was identified as a contributing factor that played a crucial role in accelerating hemostasis as a coagulation factor. Additioanlly, QCSMA/DAMA/Zn-nWH exhibited outstanding antibacterial properties with an efficacy exceeding 90 % against both *S. aureus* and *E. coli* and also demonstrated great effects on wound healing. It displayed the capability to enhance collagen deposition, reduce inflammatory expression, and actively facilitate the overall wound healing process.

In another relevant study [153], it was demonstrated that incorporating whitlockite nanoparticles (nWH) into a Ch hydrogel through a basic regeneration chemistry, pH adjustment, following by achieving uniform nWH distribution with a nanoparticle size of 75 ± 5 nm, resulted in an injectable shear-thinning hydrogel with improved hemostatic potential, elastic modulus, and biological properties in contrast to Ch hydrogel. The composite hydrogel, formulated with 2 % chitosan and 4 % nano wood hydroxyapatite, exhibited that the addition of WH influenced cytocompatibility, while an increase in nWH concentration (>4 %) decreased cell viability. Moreover, augmenting the percentage of nWH particles in the 2 % Ch hydrogel and subjecting the composite hydrogel to incubation for ion release significantly decreased the clotting time. The clotting time was reduced from 7.2 ± 0.5 min (similar to normal human whole blood clotting time) to 3.5 ± 1.2 min. This observed effect can be attributed to the synergistic action of Ca^2+^, Mg^2+^, and phosphate (PO_4_^3−^) ions released from nWH, along with the amine groups of the Ch hydrogel. The complex interplay of these components contributes to the enhanced hemostatic properties of the composite hydrogel, showing its potential for applications in wound healing and blood clotting interventions. In an *in vivo* hemostasis investigation conducted on injuries induced in the liver and femoral artery of rats, the 2 % Ch-4% nWH composite hydrogel demonstrated a reduction in hemostatic time and blood loss mass (62 ± 3 s, 478 ± 8 mg for liver injury, and 229 ± 9 s, 247 ± 10 mg for femoral artery) compared to the 2 % Ch hydrogel (93 ± 3 s, 552 ± 9 mg for liver injury and 323 ± 12 s, 337 ± 11 mg for femoral artery). Some other studies about the use of calcium-phosphate ceramics in wound healing are given in Table 4.

**Table 4:** Overview of selected studies exploring the application of calcium-phosphate ceramics in wound healing.

Bioceramic System	Bioceramic type	Description	Result	Ref
Calcium-phosphates	Bioglass	Electrospun chitosan/PVA/bioglass nanofibrous membrane	membranes showed to be effective in healing diabetics; significant increase in the expression of VEGF and TGF-β good spreading and thus stimulating angiogenesis and tissue generation	[154]
		borate based bioglass micro-fibers	bioglass samples enhanced the formation of blood vessel, showed non-toxicity against human umbilical vein endothelial cells (HUVECs) and the *in vivo* good reconstruction ability	[145]
		cerium-doped phosphate glass fibers	the antibacterial activity and controlled release of the ions is improved with doped cerium, and also sample allowed the proliferation of human epidermal keratinocyte (HaCaT) cells and the migration of mice embryonic fibroblasts (MEF); high water uptake (up to 800 %) with improved in mechanical properties in comparison to phosphate glass fibers without cerium	[155]
		Bioglass activated fibroblast sheet was prepared for wound treatment	By using the prepared construct, blood vessel formation, collagen I synthesis, differentiation of fibroblasts into myofibroblasts and wound healing were facilitated and promoted.	[143]
	Hydroxyapatite	hydroxyapatite (HA)-containing alginate–gelatin films	The incorporation of HA caused the thermal stability of films; led to a rougher surface; decreased the release rate of tetracycline hydrochloride	[156]
	Whitlockite	Injectable nanowhitlockite incorporated chitosan hydrogel was prepared for effective hemostasis	Rapid blood clot formation and bleeding control was occurred on rat liver and femoral artery injuries model.	[153]
	Whitlockite	Methacrylate anhydride dopamine and Zn-doped whitlockite nanoparticles incorporated methacrylate anhydride quaternized chitosan hydrogel was prepared for hemostasis, disinfection and healing of wounds	Excellent *in vivo* and *in vitro* hemostatic effect, antibacterial activity, and collagen deposition was achieved using the prepared nanocomposite hydrogel.	[152]

Table 5 provides a general comparison between the performance of different bioceramics in wound treatment according to the previously mentioned studies. Oxide ceramics typically release only one type of inorganic ion and their effect is often summed up in anti-inflammatory and antibacterial properties. Calcium-phosphate ceramics release more inorganic ions and some effective ions can be doped in the structure of these bioceramics. With a general point of view, it seems that silicate-based ceramics have the most performance and impact in wound healing due to the variety of effective releasing ions, and therefore they can be introduced as more perfect bioceramics for wound healing.

**Table 5:** Overall comparison of different bioceramics in terms of effect on wound healing.

Bioceramic type	Common main effective features
Oxide ceramics	- Antibacterial, antioxidant and anti-inflammatory activity
Calcium-phosphates	- Angiogenesis stimulation, hemostatic activity, cell stimulation
Silicate-based ceramics	- Antibacterial, antioxidant and anti-inflammatory activity - Stimulation of cell proliferation, angiogenesis and collagen synthesis

## 5 Conclusion and outlook

Non-healing chronic wounds represent a global crisis that imposes significant economic and social costs on healthcare systems worldwide. Countless studies have been accomplished on the use of different biomaterials for wound treatment. Polymer materials are an integral part of dressings which serve as some of the most accessible and appropriate wound healing products. Additionally, growth factors are utilized to stimulate the growth and proliferation of cells, however, they are usually expensive and have a short half-life. On the other hand, the conventional medicines and antibiotics are usually associated with side effects and the risk of bacterial resistance.

Bioceramics are commonly recognized for their robust mechanical properties and are often employed as bone repair materials or substitutes. Despite this association, their overlooked potential lies in their ability to release diverse inorganic ions, making bioceramics a valuable and economical option for effective skin repair. Herein, we highlighted the application of three main categories of nanobioceramics, i.e., oxide ceramics, silicate-based ceramics, and calcium-phosphate ceramics, in wound treatment, along with an overview of previous studies. Overall, oxide ceramics are often used due to their antibacterial and anti-inflammatory activity, while calcium-phosphate ceramics mostly stimulate hemostatic activity and angiogenesis. It is worth noting that it is possible to dope some effective ions into the structure of calcium-phosphate ceramics and develop their performance. Silicate-based ceramics contain a greater variety of ions, which widens their range of activity. In addition to antibacterial and anti-inflammatory activity, these bioceramics can play a significant role in inducing angiogenesis and promoting cell activity, thereby accelerating wound healing. Therefore, silicate-based ceramics can be introduced as a more perfect candidate in wound healing applications.

The most challenging cases in the application of bioceramics in wound healing are the synthesis methods and their scalability. Usually, the synthesis methods that lead to the production of bioceramics with high purity, have low efficiency and scaling it up may be arduous. However, even the high-cost methods of bioceramic synthesis are more economical than the methods of purification and extraction of pharmaceuticals and synthesis of growth factors.

Although promising results have been obtained from the use of bioceramics in wound treatment, aspects such as the effect of shape, size, concentration, and duration of use required further investigation. Also, there are few clinical studies in this field in the literature, which makes the final decision doubtful. Clinical studies can remove the ambiguous points of *in vitro* and even *in vivo* studies and reveal the shortcomings of the proposed methods. Fabricating nano-bioceramics using contemporary technologies like microfluidic techniques is one avenue. Another involves preparing polymer-bioceramic nanocomposite dressings, nanofibers embedded with nano-bioceramics, customized 3D-printed scaffolds, and microneedles incorporating nano-bioceramics.

These advancements, supported by comprehensive *in vitro*, *in vivo*, and clinical evaluations, have the potential to pave the way for more effective and economically viable approaches to wound healing in the future. The integration of cutting-edge technologies and rigorous testing not only enhances the current understanding of wound healing mechanisms but also promises innovative solutions for improved patient outcomes and cost efficiency.

## Ethical approval

Not Applicable.

## Data and code availability

Data will be made available on request.

## CRediT authorship contribution statement

**Hanan Adnan Shaker Al-Naymi:** Writing – original draft, Validation, Data curation. **Mastafa H. Al-Musawi:** Writing – original draft, Validation, Software, Data curation. **Marjan Mirhaj:** Writing – review & editing, Writing – original draft, Visualization, Validation, Supervision, Software, Methodology, Investigation, Formal analysis, Data curation, Conceptualization. **Hamideh Valizadeh:** Writing – original draft, Validation, Methodology, Formal analysis, Data curation. **Arefeh Momeni:** Visualization, Validation, Formal analysis, Data curation. **Amir Mohammad Danesh Pajooh:** Writing – original draft, Visualization, Formal analysis, Data curation. **Mina Shahriari-Khalaji:** Writing – original draft, Validation, Investigation, Formal analysis, Data curation. **Fariborz Sharifianjazi:** Writing – original draft, Visualization, Validation, Formal analysis, Data curation. **Ketevan Tavamaishvili:** Writing – original draft, Data curation. **Nafise Kazemi:** Writing – original draft, Validation, Funding acquisition, Data curation. **Saeideh Salehi:** Writing – original draft, Visualization, Formal analysis, Data curation. **Ahmadreza Arefpour:** Writing – original draft, Visualization, Validation, Formal analysis, Data curation. **Mohamadreza Tavakoli:** Writing – review & editing, Writing – original draft, Visualization, Validation, Supervision, Software, Project administration, Formal analysis, Data curation, Conceptualization.

## Declaration of competing interest

The authors declare that they have no known competing financial interests or personal relationships that could have appeared to influence the work reported in this paper.

## Abbreviations

45S5 Bioglass®: (SiG)
Aluminum: (Al)
Aluminum oxide: (Al_2_O_3_)
Aluminum-oxide membrane: (AAO)
Ampicillin: (Amp)
Basic fibroblast growth factor: (bFGF)
Bio-glasses: (BG)
Borate bioactive glass: (bBG)
Bredigite: (BR)
Carboxymethyl chitosan: (CMC)
Calcium: (Ca)
Chick chorioallantoic membrane: (CAM)
Chitosan: (Ch)
Chitosan/gelatin: (CG)
Cirsium setidens extract: (CSE)
Copper: (Cu)
Copper exchanged faujasite: (CAF)
Copper oxide: (CuO)
Copper oxide microparticles: (COD)
Diamond-like carbon: (DLC)
Endothelial nitric oxide synthase: (eNOS)
Epidermis growth factor: (EGF)
Erythromycin: (Ery)
Extracellular matrix: (ECM)
*Escherichia coli*: (*E. coli*)
Fibrinogen: (FG)
Focal adhesion kinase: (FAK)
Gellan Gum: (GG)
HA nanowires-based biopaper reinforced with a small number of cellulose fibers: (HAPNW/CF biopaper)
Hardstonite: (ZnCS)
Human dermal fibroblasts: (HDFs)
Human epidermal stem cells: (hESCs)
Hydroxyapatite: (HA)
Mesoporous SiO_2_ nanoparticles: (MSNs)
Methacrylate anhydride dopamine: (DAMA)
Methacrylate anhydride quaternized chitosan: (QCSMA)
Oxygen: (O)
Poly (3-hydroxybutyrate-co-3-hydroxyvalerate): (PHBV)
Poly (vinyl alcohol): (PVA)
Polycaprolactone: (PCL)
Polyelectrolyte complex: (PEC)
Polylactic acid: (PLA)
Reactive oxygen species: (ROS)
Red blood cells: (RBC)
Silicon: (Si)
Silicon oxide: (SiO_2_)
Silver: (Ag)
Sliver Oxide nanoparticles: (Ag_2_O NPs)
Sodium alginate: (SA)
*Staphylococcus aureus*: (*S. aureus*)
Phosphorus: (P)
Tetrafluoroethylene: (TPE)
The clove extract: (CE)
TiO_2_ nanostructures: (TNSs)
Titanium: (Ti)
Titanium dioxide: (TiO_2_)
Titanium Dioxide Nanotubes: (NTs)
TiO_2_ nanotubes: (TNTs)
Vascular endothelial growth factor A: (VEGFA)
Whitlockite: (WH)
Whitlockite nanoparticles: (nWH)
Zinc: (Zn)
Zinc-doped Wollastonite: (Zn-Ws)
Zinc oxide nanoparticle: (ZnO NPs)
Zinc-doped bioactive glass: (Gel-ZBG)
Zirconium: (Zr)
zirconium dioxide nanoparticles: (ZrO_2_ NPs)
Zirconium oxide: (ZrO_2_)
Zn-doped whitlockite nanoparticles: (Zn-nWH)
