# Supplementary material for: Exploring nanobioceramics in wound healing as effective and economical alternatives
Source: Heliyon. 2024 Sep 26;10(19):e38497. doi: 10.1016/j.heliyon.2024.e38497 (PMC11466581; doi:10.1016/j.heliyon.2024.e38497)
Supplement: Multimedia component 1 [file mmc1.docx]

Table S1. The price list of some important bioceramics precursors, as well as some of the most important pure pharmaceutical active ingredients and growth factors.

| Basic substance | Precursor/active ingredient | Amount | Price (USD) | Manufacturer company |
| --- | --- | --- | --- | --- |
| Bioceramic | Tetraethyl orthosilicate (TEOS) (Si(OC₂H₅)₄) | 1 mL | 0.12 | Sigma-Aldrich |
|  | Zirconium (IV) nitrate hexahydrate (ZrO(NO_3_)_2_⋅6H_2_O) | 1 g | 1.37 | Sigma-Aldrich |
|  | Calcium nitrate tetrahydrate (Ca(NO_3_)_2_⋅4H_2_O) | 1 g | 0.13 | Sigma-Aldrich |
|  | Zinc nitrate hexahydrate (Zn(NO_3_)_2_.6H_2_O) | 1 g | 0.08 | Sigma-Aldrich |
|  | Magnesium nitrate hexahydrate (Mg (NO_3_)_2_·6H_2_O) | 1 g | 0.40 | Sigma-Aldrich |
|  | Phosphorus pentoxide (P_2_O_5_) | 1 g | 0.19 | Sigma-Aldrich |
|  | Silver nitrate (AgNO_3_) | 1 g | 5.85 | Sigma-Aldrich |
|  | Strontium nitrate (Sr(NO_3_)_2_) | 1 g | 0.47 | Sigma-Aldrich |
|  | Diammonium hydrogen phosphate  ((NH_4_)_2_HPO_4_) | 1 g | 0.16 | Sigma-Aldrich |
|  | Copper nitrate (Cu(NO_3_)_2_ | 1 g | 3.21 | Sigma-Aldrich |
| Growth factor | IGF1 | 1 µg | 6.57 | Sigma-Aldrich |
|  | VEGF | 1 µg | 51.15 | Sigma-Aldrich |
|  | TGF-ß | 1 µg | 243.65 | Sigma-Aldrich |
|  | PDGF-AB | 1 µg | 51.15 | Sigma-Aldrich |
| Active pharmaceutical ingredient | Gentamicin sulfate | 1 mg | 1.08 | Sigma-Aldrich |
|  | Vancomycin hydrochloride | 1 mg | 0.77 | Sigma-Aldrich |
|  | Mupirocin | 1 mg | 11.24 | Sigma-Aldrich |
|  | Simvastatin | 1 mg | 13.28 | Sigma-Aldrich |
|  | Deferoxamine Mesylate | 1 mg | 6.05 | Sigma-Aldrich |
|  | Tetracycline | 1 g | 6.40 | Sigma-Aldrich |
|  | Ciprofloxacin | 1 g | 6.66 | Sigma-Aldrich |
